# Manipulation of DHPS activity affects dendritic morphology and expression of synaptic proteins in primary rat cortical neurons

**DOI:** 10.3389/fncel.2024.1465011

**Published:** 2024-10-14

**Authors:** Paola Cavalli, Anna Raffauf, Sergio Passarella, Martin Helmuth, Daniela C. Dieterich, Peter Landgraf

**Affiliations:** ^1^Institute for Pharmacology and Toxicology, Otto von Guericke University Magdeburg, Magdeburg, Germany; ^2^Center for Behavioral Brain Sciences (CBBS), Magdeburg, Germany

**Keywords:** DHPS, eIF5A hypusination, neuron, synapse, homeostasis

## Abstract

Deoxyhypusine synthase (DHPS) catalyzes the initial step of hypusine incorporation into the eukaryotic initiation factor 5A (eIF5A), leading to its activation. The activated eIF5A, in turn, plays a key role in regulating the protein translation of selected mRNAs and therefore appears to be a suitable target for therapeutic intervention strategies. In the present study, we analyzed the role of DHPS-mediated hypusination in regulating neuronal homeostasis using lentivirus-based gain and loss of function experiments in primary cortical cultures from rats. This model allows us to examine the impact of DHPS function on the composition of the dendritic and synaptic compartments, which may contribute to a better understanding of cognitive function and neurodevelopment *in vivo*. Our findings revealed that shRNA-mediated DHPS knockdown diminishes the amount of hypusinated eIF5A (eIF5A^Hyp^), resulting in notable alterations in neuronal dendritic architecture. Furthermore, in neurons, the synaptic composition was also affected, showing both pre- and post-synaptic changes, while the overexpression of DHPS had only a minor impact. Therefore, we hypothesize that interfering with the eIF5A hypusination caused by reduced DHPS activity impairs neuronal and synaptic homeostasis.

## 1 Introduction

Deoxyhypusine synthase (DHPS) plays a crucial role in the hypusination of the eukaryotic initiation factor 5A involved in protein synthesis, cellular growth, and function (Park, 2006). The enzyme catalyzes the first step of this unique posttranslational modification, using the 4-aminobutyl spermidine moiety to form the deoxyhypusine intermediate on its residue Lys50 (rat/human protein number). Although much is already known about the DHPS-mediated eIF5A^Hyp^ in other cell types (Park et al., 1993; Park et al., 1998; Park et al., 2010), the significance of this process in neurons has been little investigated. Previous experiments have shown that impaired growth and survival in PC12 neuronal cultures correlate with dysfunctional hypusination (Huang et al., 2007). Furthermore, impairment in cognitive functions was demonstrated in DHPS-KO and eIF5A-KO-based mouse models, suggesting the function of DHPS/eIF5A in neurological development (Kar et al., 2021). In this context, the role of DHPS/eIF5A in pathological conditions remains poorly investigated. Thus, *in vitro* and *in vivo* models would be useful, as demonstrated in studies for human diseases associated with recessive rare variants of DHPS or immunopathogenesis of type 1 diabetes (Colvin et al., 2013; Ganapathi et al., 2019). However, there is limited research that directly links DHPS to neuronal homeostasis and synaptic functionality.

Nonetheless, the involvement of eIF5A in the biosynthesis of specific proteins suggests that DHPS may exert an indirect effect on synaptic plasticity and neurotransmission via the modulation of eIF5A activity. These proteins are primary proteins with polyproline-rich motifs, in which eIF5A^Hyp^ promotes translation and prevents the formation of stalled ribosomes (Gutierrez et al., 2013; Doerfel et al., 2013) and are also found in both post- and pre-synapses (Pielot et al., 2012; Cajigas et al., 2012; Suzuki et al., 2007). Indeed, DHPS might indirectly influence the production of proteins that are critical for maintaining the cytoskeletal structure of neurons, including microtubules and actin filaments, which are involved in axons and dendrite growth, dendritic spine formation, and neuronal synaptic plasticity (Leuner and Gould, 2010; Runge et al., 2020). Notably, research using zebrafish models has delineated the human phenotype associated with eIF5A knockdown, typified by Mendelian disorders. These disorders have been demonstrated to be ameliorable through the administration of 1 μM spermidine supplementation, thereby elucidating the role of eIF5A in human pathology and propelling therapeutic prospects (Faundes et al., 2021). In this context, it would be of particular interest to decipher to what extent DHPS activity is involved in developing and sustaining neurodegenerative diseases associated with significant disruptions in neuronal homeostasis.

In the presented report, we analyzed the effects of altered DHPS activity on neuronal homeostasis using primary cortical cultures as a model system, focusing on synapse formation and dendritic morphology. Thereby, we aimed to answer the question of whether targeted manipulation of DHPS activity can contribute to maintaining neuronal functionality and plasticity.

## 2 Results

### 2.1 eIF5A hypusination is compromised in DHPS-shRNA neural cortical cultures

**Figure 1:**
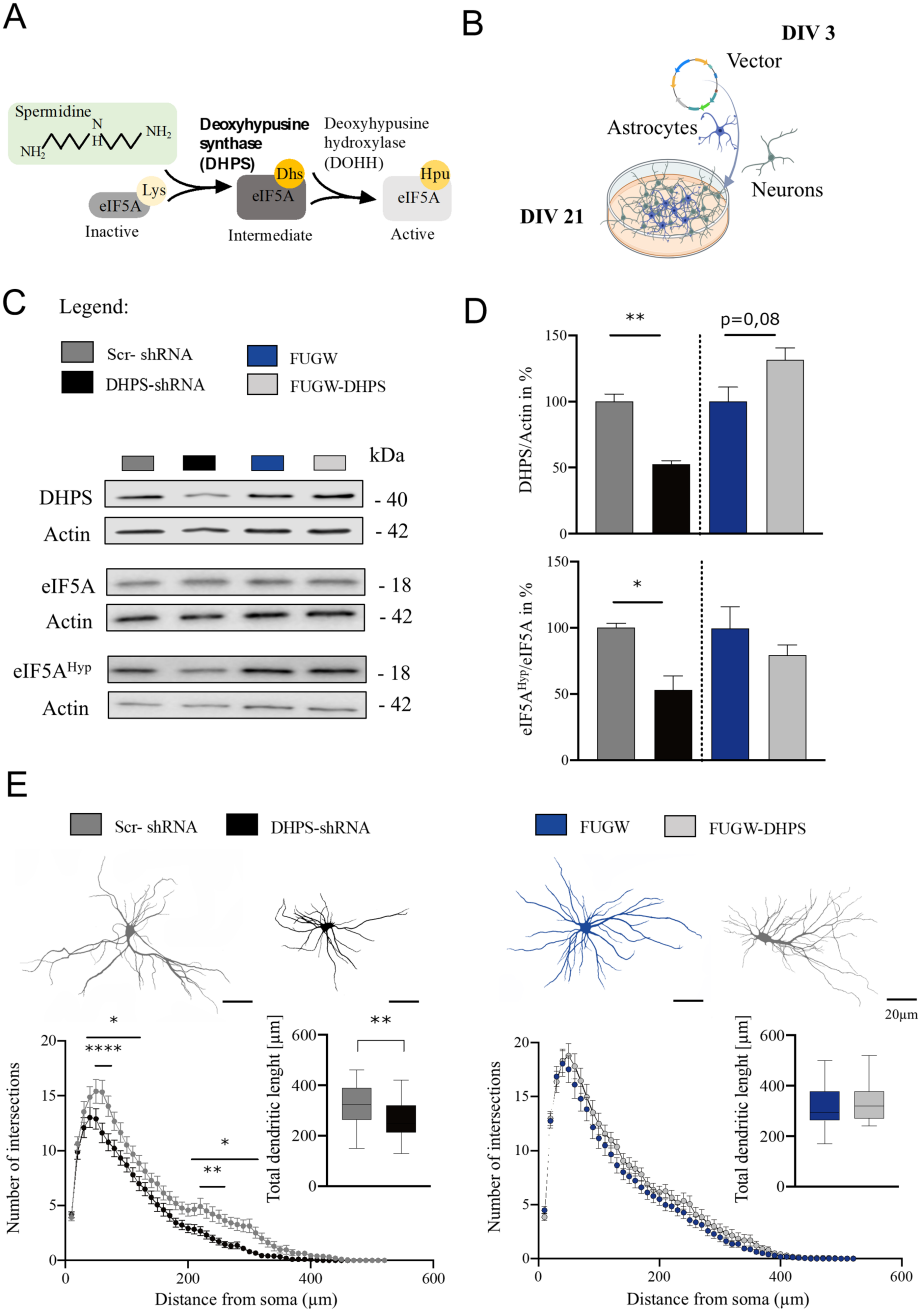
Deoxyhypusine synthase (DHPS)-mediated hypusination correlates with changes in neuronal architecture. **(A)** Graphical abstract of the eIF5A hypusination. **(B)** Experimental design of virus infection at day *in vitro* 3 (DIV 3), followed by continuous growth until DIV 21 (created with BioRender.com). **(C,D)** Representative Western blots of protein extracts from DHPS-shRNA and FUGW-DHPS expressing primary cortical cultures. Actin was used for internal normalization. Values are the mean of two to three technical replicates in each group from *N* = 3 independent biological replicates for immunoblotting analysis. **(E)** Sholl analysis was performed via immunocytochemistry using MAP2-stained primary cortical neurons at DIV21. An average of 30–45 neurons were used from three independent experiments. Scale bar 20 μm. All data are presented as the mean ± SEM. Symbols for *p*-values used in the figures: *p < 0.05, **p < 0.01, ****p < 0.0001. *p*-values were calculated using a two-tailed Student’s *t*-test for immunoblots and a two-way ANOVA followed by Tukey’s *post hoc* test for the immunocytochemistry.

Deoxyhypusine synthase is the key enzyme for synthesizing the hypusine intermediate [Nε-(4-aminobutyl) lysine]. This intermediate plays a crucial role in the hypusination and thereby the activation of eIF5A, which essentially contributes to the survival and proliferation of eukaryotic mammalian cells (Nishimura et al., 2012). To assess the activity of DHPS in mature neural cortical co-cultures at day *in vitro* 21 (DIV 21), we analyzed eIF5A^Hyp^ using Western blot and found that the hypusine content in the DHPS-shRNA expressing cultures was reduced by approximately 45% (mean = 56.36; SEM = 13.03), while the levels of eIF5A protein remained constant (Figures 1A–D). This observation may be attributed to the reduced levels of DHPS caused by its knockdown in the neural co-cultures (mean = 52.72; SEM = 6.29). In contrast, the DHPS-overexpressing group showed no effect compared to the control (Figures 1C,D).

### 2.2 Preventing hypusination causes changes in the dendritic arborization of neurons

Primary DIV21 cortical neurons expressing DHPS knockdown or overexpression constructs were stained for the microtubule-associated protein 2 (MAP2) and used to perform Sholl analysis. Our findings suggest that the inhibition of hypusination resulted in a reduction in the number of dendritic branches both proximally and distally (Figure 1E). Moreover, the total length of dendrites was significantly shortened in DHPS-shRNA expressing neurons compared to the control group (MD = 56.75; SEM = 17.92). In contrast, since DHPS overexpression in neurons does not exhibit significant morphological changes, it might suggest that neurons endogenously possess sufficient amounts of this enzyme.

### 2.3 Reduced hypusination of eIF5A affects the synaptic composition of cortical neurons

**Figure 2:**
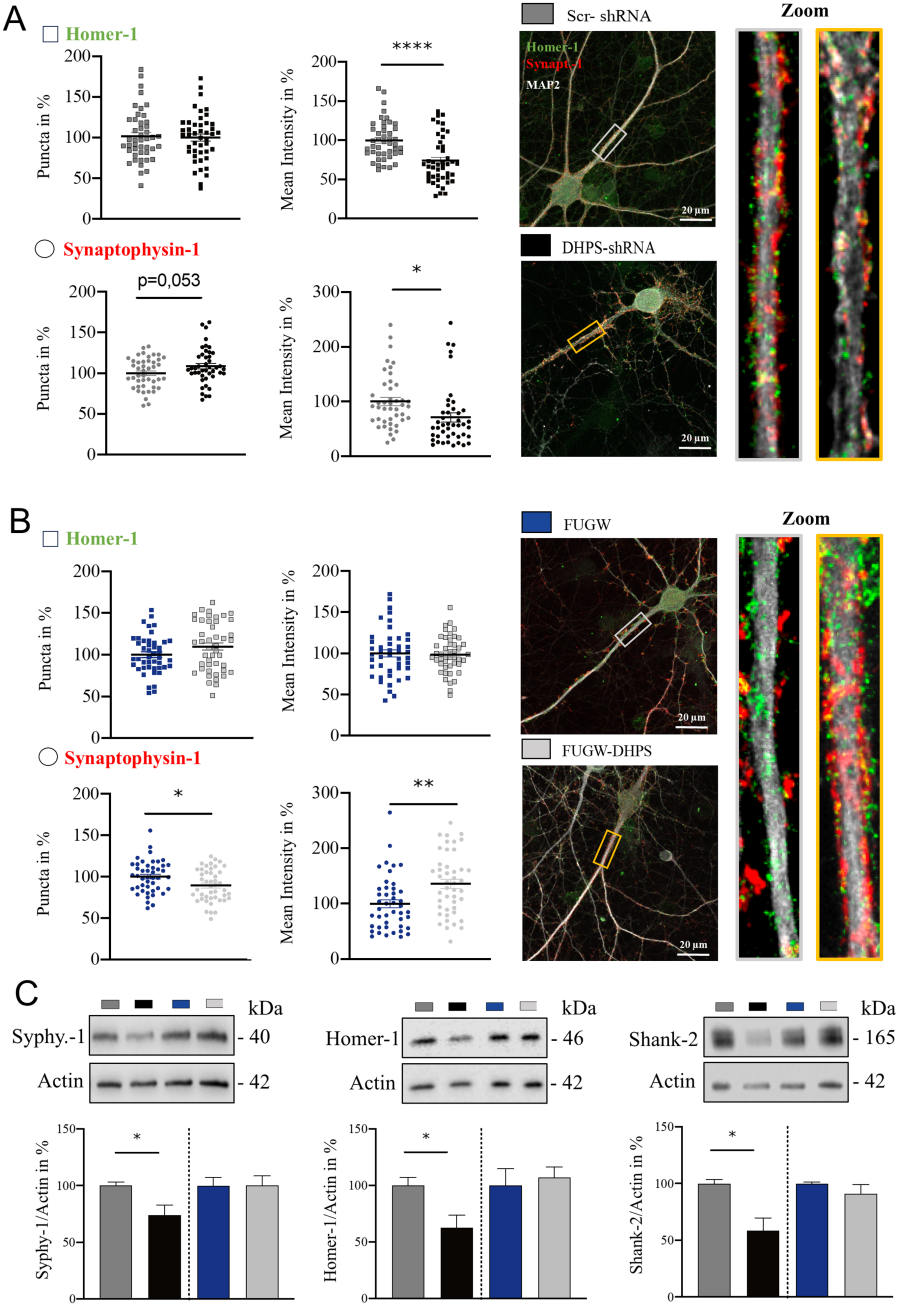
Impaired hypusination affects the level of pre- and post-synaptic proteins. **(A)** Representative confocal images of major dendrites from primary cortical neurons at DIV21 expressing DHPS-shRNA for knockdown and scrambled Scr-shRNA as a control. IF staining shows the pre-synaptic marker synaptophysin-1 (red), the post-synaptic marker Homer-1 (green), and the dendritic marker MAP2 (white). **(B)** Representative confocal images of primary cortical neurons overexpressing DHPS (DHPS-FUGW) or the empty vector (FUGW) as a control. For IF staining, the same markers were used as shown in **(A)**. The number of synaptic puncta was analyzed using ImageJ with the plugin SynQuant. An average of 45 neurons were used from three independent experiments. Synaptic puncta and size in **(A,B)** were analyzed at the respective proximal major dendrite at a length of 20 μm as depicted in the rectangle of the images and magnified presented on the right side. Scale bar 20 μm. **(C)** Representative Western blots and quantitative analysis of protein extracts from DHPS-shRNA and DHPS-FUGW expressing primary cortical cultures at DIV21 compared to cultures expressing the indicated control vectors. Actin was used as an internal control. Values are the mean of two to three technical replicates in each group from *N* = 3 independent biological replicates for immunoblotting analysis. All data are presented as the mean ± SEM. Symbols for *p*-values used in the figures: *p < 0.05, **p < 0.01, and ****p < 0.0001. *p*-values were calculated using a two-tailed Student’s *t*-test.

Modifications in the dendritic tree are not isolated events but have far-reaching impacts on synaptic connectivity, thereby playing a pivotal role in shaping neuronal function and contributing to various cognitive processes (Kaech et al., 2001). Therefore, we next analyzed whether the observed changes in cytoarchitecture were associated with pre- and post-synaptic changes. In DHPS-shRNA expressing neurons, we observed a significant decrease in Homer-1 intensity (mean = 73.94; SEM = 5.8), whereas no detectable alterations were observed in the number of Homer-1 puncta, suggesting a disruption in the composition of the post-synaptic complex (Figure 2A). Similarly, synaptophysin-1-labeled pre-synaptic structures in DHPS-shRNA expressing neurons appeared to be disrupted, as a decrease in fluorescent intensity was also observed here (mean = 70.98; SEM = 11.07; Figure 2A). In contrast, neurons overexpressing DHPS showed changes only in the pre-synaptic compartment, where we observed a decrease in the total number of synaptophysin-1 signals on the one hand (mean = 89.22; SEM = 4.11), but a significant increase of the synaptophysin-1 intensity on the other (mean = 136.20; SEM = 11.01; Figure 2B). In this context, no changes were observed at the post-synapse (Figure 2B). Additionally, we were able to confirm these findings using Western blots and showed that a knockdown of DHPS, combined with a lower eIF5A^Hyp^, affects both the post-synaptic and pre-synaptic composition (Homer-1: mean = 62.62; SEM = 12.98 and synaptophysin-1: mean = 73.97; SEM = 9.34), whereas overexpression of the enzyme did not lead to significant changes (Figure 2C). Considering the fact that hypusinated eIF5A plays a crucial role in the translation of polyproline sequences (Shin et al., 2017), we analyzed Shank-2 harboring two proline-rich domains as a respective candidate using Western blot (Figure 2C). The results obtained in this context underline that, on the one hand, downregulation of DHPS results in a significant reduction in the expression of synaptic proteins (mean = 58.36; SEM = 11.81), while, on the other hand, overexpression of DHPS has no consequences for their expression (Figure 2C). In conclusion, our data demonstrate that a deficiency in eIF5A^Hyp^ caused by downregulated DHPS level negatively impacts synaptic proteostasis. In contrast, overexpression of DHPS neither leads to increased eIF5A^Hyp^ nor a significant influence on synaptic proteostasis, indicating a saturated capacity of the endogenous DHPS.

## 3 Discussion

In humans, the rare autosomal recessive inherited disorder of DHPS deficiency is associated with various neurological impairments, including seizures, developmental delay, and intellectual disability (Padgett et al., 2023; Ganapathi et al., 2019). Therefore, in recent years, scientific research has increasingly focused on the importance of the DHPS/eIF5A pathway for neuronal development and functioning, but many functions are still elusive. Nevertheless, in this context, it could be shown that both DHPS and eIF5A^Hyp^ depletion are detrimental or lethal for mouse embryos (Ganapathi et al., 2019; Kar et al., 2021). Using different genetic mouse models depleted for DHPS or eIF5A in specific areas of the brain, Kar et al. (2021) could show an impairment in neuronal growth, viability, neurodevelopment, and cognitive function. Moreover, eIF5A dysfunction has been linked to Mendelian disorders marked by a spectrum of developmental anomalies (Faundes et al., 2021). Interestingly, Huang et al. (2007) demonstrated the impact of a disturbed DHPS/eIF5A axis on primary hippocampal neurons. They were able to show that blocking DHPS activity by GC7 leads to a significantly reduced dendrite length of neurons that can be attributed to thereby decreased levels of hypusinated eIF5A.

In the present study, we aimed to analyze the impact of DHPS manipulations at the neuronal level and used primary cortical neuron-astrocyte co-cultures from rats as an *in vitro* model system in order to analyze the effect of both Lentivirus-mediated DHPS overexpression and depletion on dendritic morphology and changes in the levels of selected synaptic proteins contributing to the synaptic plasticity of neurons. Thereby, we could show that the depletion of DHPS significantly reduces the level of hypusinated eIF5A in the primary cultures, whereas its overexpression had no impact on the content of eIF5A^Hyp^, pointing to the fact that the DHPS activity seems to be saturated under steady-state conditions. The downregulation of eIF5A^Hyp^ caused by DHPS knockdown is directly linked to alterations of the neuronal morphology that become detectable by a reduction of dendritic length and complexity, whereas unchanged eIF5A^Hyp^ level after DHPS overexpression does not alter neuronal morphology. We therefore hypothesized that neuronal architecture directly depends on the functional hypusination of eIF5A, as supposed by Huang et al. (2007). Furthermore, our finding is consistent with reports from Kar et al. (2021) who have shown significant developmental and morphological impairments in various brain areas of DHPS or eIF5A-deficient mice, resulting in reduced cognitive functions and viability. Additionally, our findings showed a reduction in the levels of pre- and post-synaptic proteins in DHPS-deficient neurons, pointing toward impaired structural synaptic plasticity that has to be verified in continued experiments. In this context, a direct correlation with the DHPS-dependent eIF5A^Hyp^ is particularly evident for the post-synaptic scaffold protein Shank-2, which contains multiple proline-rich motifs, aligning with the proposed function of eIF5A in the translation of polyproline-containing proteins (Saini et al., 2009; Gutierrez et al., 2013; Mandal et al., 2014). This observation fits with reports from Doerfel et al. (2013) and Gutierrez et al. (2013), demonstrating a strong correlation between decreased eIF5A hypusination and increasing amounts of stalled ribosomes leading to reduced protein translation. A reduced protein synthesis caused by decreased DHPS activity and eIF5A hypusination was also reported by Padgett et al. (2023). Using quantitative mass spectrometry analysis, they found an altered expression of proteins directly involved in dendrite extension, synapse formation, neurotransmission, and neuronal membrane projection and secretion (Padgett et al., 2023). However, further analyses would be necessary to make a more specific statement. Interestingly, in neurons overexpressing DHPS, we observed alterations in pre-synaptic structures. Although the total amounts of synaptophysin-1 remained unchanged, immunofluorescence microscopy revealed a reduction in the number of synaptophysin-1-positive presynapses, but a significant increase in signal intensity. Notably, since we did not detect elevated levels of elF5A^Hyp^ in DHPS overexpressing neurons, this phenomenon requires further investigation and might be independent of eIF5A hypusination.

Although we have demonstrated a direct association between DHPS-dependent hypusination of eIF5A and morphological changes of neurons as well as the impaired synthesis of pre- and post-synaptic proteins, this study has several limitations. For example, a more detailed analysis of synaptic structural changes as well as their significance for functional changes in synaptic transmission would be of great importance. Nevertheless, we believe that our report represents a significant contribution to the understanding of DHPS/eIF5A^Hyp^ activity-mediated influences on neuronal morphology and function and thus lays the foundation for further investigations using the presented *in vitro* model.

## 4 Methods

### 4.1 Animals

The experiments were approved by the local Ethics Commission of the Federal State of Saxony-Anhalt (according to the European Communities Council Directive; 86/609/EEC) and were conducted in accordance with European regulations for ethical care and use of laboratory animals (2010/63/EU). Adult Wistar rats (*Rattus norvegicus*) were housed in groups of 5–6 animals, under regular 12 h light–dark cycles (lights on 6 a.m.–6 p.m.) with food and water available *ad libitum* at constant temperature (22 ± 2°C) and air humidity (40–60%). Efforts were made to minimize the number of animals used and to reduce their suffering during experiments.

### 4.2 Primary neural cultures from the rat cortex

Primary rat cortical cells were obtained from Wistar rats at embryonic days 18–19 (E18.5). Pregnant rats were anesthetized with isofluran (Baxter GmbH) for 2 min prior to euthanasia. The E18.5 embryos’ brains were dissected and prepared as described by Müller et al. (2015). After mechanical and enzymatical disassociation of the cortices, the cells were seeded in DMEM supplemented with 10% FBS (Gibco), 100 U/mL of penicillin (Gibco), 100 μg/mL of streptomycin (Gibco), and 2 mM L-glutamine (Gibco) onto poly-D-lysine (Gibco) pre-coated well plates (Techno Plastic Product-TPP). After 3 and 24 h, the medium was replaced with Neurobasal™ medium (Gibco) supplemented with 2% B27 (Gibco) and 0.8 mM L-glutamine (Gibco). The cells were fed weekly with Neurobasal™ by 10% of the total volume. The cultures were kept in an incubator constantly at 37°C and 5% CO_2_. For Western blot experiments, the cells were seeded in 6-well plates at a density of 300.000 cells/well (TPP). For immunocytochemistry, they were seeded in 12-well plates (TPP) at a density of 40.000 cells/well.

### 4.3 Vector construction and lentivirus generation

The plasmid pFUGW was a gift from David Baltimore (Addgene plasmid # 14883; http://n2t.net/addgene:14883; RRID:Addgene_14,883); and the plasmids FUGW-H1 and FUGW-H1-Scrambled were gifts from Sally Temple (Addgene plasmid # 25870; http://n2t.net/addgene:25870; RRID:Addgene_25870 and Addgene plasmid # 40625; http://n2t.net/addgene:40625; RRID:Addgene_40,625). For overexpressing DHPS, the entire ORF of the cDNA from rats (GenBank-ID: NM_001004207.1) was cloned into the FUGW vector and expressed under the control of the ubiquitin promoter. For the knockdown of DHPS, sh-oligos were inserted into the FUGW-H1 vector according to the provided cloning protocol, and the shRNA expression cassette was driven by the H1 promoter. The used DHPS-shRNA sequence was: GCTATACGTCCAACCTCAT. For packing the vectors into Lentiviruses, the packing plasmid psPAX2 (a gift from Didier Trono Addgene plasmid # 12260; http://n2t.net/addgene:12260; RRID:Addgene_12,260) and the plasmid for the envelope protein pCMV-VSV-G [a gift from Bob Weinberg (Addgene plasmid # 8454; http://n2t.net/addgene:8454; RRID:Addgene_8,454)] were used. The preparation of lentiviruses was conducted as previously published (Müller et al., 2015). For the knockdown experiments, the FUGW-H1-DHPS-shRNA vector (DHPS-shRNA) was used with the FUGW-H1-Scrambled vector (Scr-shRNA) as a control. For the over-expression experiments, the FUGW vector containing the DHPS ORF (FUGW-DHPS) was used with the empty FUGW vector (FUGW) as a control.

### 4.4 Immunocytochemistry

DIV 21 cultures were briefly rinsed with 1X PBS, pH 7.4, containing 1 mM MgCl_2_ and 0.1 mM CaCl_2_ and fixed with periodate-lysine-paraformaldehyde for 30 min at RT (McLean and Nakane, 1974). Then, the coverslips were washed three times in 1X PBS pH 7.4 for 10 min each, incubated in blocking solution (B-Block) containing 10% (w/v) normal horse serum, 5% (w/v) sucrose, 2% (w/v) bovine serum albumin, and 0.2% (v/v) Triton X-100 in PBS, pH 7.4 for 1 h at RT, and finally mounted in 10% Mowiol (Carl Roth, 0713.1) and stained with primary antibodies diluted in B-Block O.N. at 4°C. The primary antibodies were: rabbit anti-Homer-1 (SySy, 160,003, 1:1000), guinea pig anti-synaptophysin-1 (SySy, 101,004, 1:1000), and mouse anti-MAP2 (Sigma, M4403, 1:1000). On the next day, the samples were washed three times in 1X PBS pH 7.4 for 10 min each and subsequently stained with the indicated Alexa Fluor dye-conjugated secondary antibodies (from Invitrogen and Jackson Immunoresearch Europe) diluted in B-Block for 1 h at RT. Finally, the coverslips were rinsed, mounted with Mowiol (Calbiochem^TM^), and imaged using confocal laser scanning microscopy (CLSM; Axio observer Z1 microscope provided with an LSM 710 confocal system; Carl Zeiss group) for synapse evaluation and a fluorescence microscope (Leica DM6 B LED) for Sholl analysis.

### 4.5 Synapse imaging and Sholl analysis

Pre- and post-synapses were labeled as described in Chapter 4.4 and evaluated as maximum intensity projection (89.97×89.97 μm–1024 × 1024 pixels) using the ImageJ plugin SynQuant (Wang et al., 2020). Images were taken in Z-stacks with 8-bit and 0.08 μm pixel size using a Plan-Apochromat 63x/numerical aperture 1.4 oil DIC M27 immersion objective, and identical exposure times were used for the quantification of all experimental groups. MAP2 signal was used as a mask to create a surface for quantifying the synapses puncta and their respective mean intensity. For the Sholl analysis, images were captured using objective HC PL Apo 20x/0.80 WD 0.4 mm and measured using the ImageJ plug-in Simple Neurite Tracer (Arshadi et al., 2021).

### 4.6 SDS-PAGE and Western blot

The cultures were lysed using protein sample buffer (62.5 mM Tris–HCl, pH 6.8, 1% SDS, 10% glycerol, 5% ß-mercaptoethanol, 0.001% bromophenol blue), incubated at 95°C for 5 min, cooled on ice, and stored at –20°C. Protein concentrations were estimated using the amido black assay and adjusted to 1 μg/μl (Schaffner and Weissmann, 1973). For each sample, 10 μg total protein was loaded on 5–20% SDS-polyacrylamide gels and separated at a current of 12 mA/gel, maintained at a constant temperature of 4°C (Laemmli, 1970). Subsequently, proteins were transferred to nitrocellulose (NC) membranes (Protran BA85, 0.22 μm, LI-COR Biosciences) using the Hoefer™ Mighty Small System SE250 (Fisher Scientific) at 200 mA for 90 min at a constant temperature of 4°C. After Ponceau staining, the membranes were blocked for 1 h at RT in a blocking solution (1xTBS, pH 7.4, containing 0.1% Tween 20 and 5% dry milk) and finally incubated O.N. at 4°C with 1xTBS, pH 7.4, containing 0.1% Tween 20 and the respective primary antibody. The next day, the membranes were washed three times with 1xTBS, pH 7.4, containing 0.1% Tween 20. Finally, the membranes were incubated for 1 h at RT in a blocking solution containing peroxidase-conjugated secondary antibodies (Dianova), washed again three times with 1xTBS, pH 7.4, containing 0.1% Tween 20 and finally imaged in a LICOR OdysseyFC (LI-COR) using ECL-solution according to the manufacturer’s protocol (Pierce™ ECL, Thermo Fisher). Primary antibodies used were: mouse/rabbit anti-Actin (CST, 8457S/3700S, 1:2000), mouse anti-DHPS (Santa Cruz, sc-376580, 1:1000), mouse anti-eIF5A (BD, 611977, 1:1000), rabbit anti-Hypusine (Merk EDM Millipore, ABS1064-I-100UL, 1:1000), rabbit anti-Homer-1 (SySy, 160,003, 1:1000), guinea pig anti-synaptophysin-1 (SySy, 101,004, 1:1000), and guinea pig anti-Shank-2 (SySy, 162,204, 1:1000). All images and quantifications were conducted using Image Studio ™ software and Licor Odyssey^FC^ (LI-COR).

### 4.7 Statistical analyses

All statistical analyses were performed using GraphPad Prism software (v.8). Repeated measurement two-way ANOVA followed by the Holm–Sídák test was conducted for Sholl analysis in neurons. A two-tailed *t*-test was applied to quantitative immunoblotting, synaptic puncta, and intensity measurements. The data are shown as mean ± standard error of the mean (SEM). Statistical significance was declared as a *p*-value of ≤0.05 for all the datasets.

## Data Availability

The datasets presented in this study can be found in online repositories. The names of the repository/repositories and accession number(s) can be found: https://www.ncbi.nlm.nih.gov/, NM_001004207.1; http://n2t.net/addgene:14883, RRID:Addgene_14883; http://n2t.net/addgene:25870, RRID:Addgene_25870; http://n2t.net/addgene:40625, RRID:Addgene_40625; http://n2t.net/addgene:12260, RRID:Addgene_12260; http://n2t.net/addgene:8454, RRID:Addgene_8454.

## References

[ref1] Arshadi C. Günther U. Eddison M. Harrington K. I. S. Ferreira T. A. (2021). SNT: a unifying toolbox for quantification of neuronal anatomy. Nat. Methods 18, 374–377. doi: 10.1038/s41592-021-01105-7, PMID: 33795878

[ref2] Cajigas I. J. Tushev G. Will T. J. tom Dieck S. Fuerst N. Schuman E. M. (2012). The local transcriptome in the synaptic neuropil revealed by deep sequencing and high-resolution imaging. Neuron 74, 453–466. doi: 10.1016/j.neuron.2012.02.036, PMID: 22578497 PMC3627340

[ref4] Colvin S. C. Maier B. Morris D. L. Tersey S. A. Mirmira R. G. (2013). Deoxyhypusine synthase promotes differentiation and proliferation of T helper type 1 (Th1) cells in autoimmune diabetes*. J. Biol. Chem. 288, 36226–36235. doi: 10.1074/jbc.M113.473942, PMID: 24196968 PMC3868737

[ref6] Doerfel L. K. Wohlgemuth I. Kothe C. Peske F. Urlaub H. Rodnina M. V. (2013). EF-P is essential for rapid synthesis of proteins containing consecutive proline residues. Science 339, 85–88. doi: 10.1126/science.1229017, PMID: 23239624

[ref7] Faundes V. Jennings M. D. Crilly S. Legraie S. Withers S. E. Cuvertino S.. (2021). Impaired eIF5A function causes a Mendelian disorder that is partially rescued in model systems by spermidine. Nat. Commun. 12:833. doi: 10.1038/s41467-021-21053-2, PMID: 33547280 PMC7864902

[ref8] Ganapathi M. Padgett L. R. Yamada K. Devinsky O. Willaert R. Person R.. (2019). Recessive rare variants in Deoxyhypusine synthase, an enzyme involved in the synthesis of Hypusine, are associated with a neurodevelopmental disorder. Am. J. Hum. Genet. 104, 287–298. doi: 10.1016/j.ajhg.2018.12.017, PMID: 30661771 PMC6369575

[ref9] Gutierrez E. Shin B. S. Woolstenhulme C. J. Kim J. R. Saini P. Buskirk A. R.. (2013). eIF5A promotes translation of polyproline motifs. Mol. Cell 51, 35–45. doi: 10.1016/j.molcel.2013.04.021, PMID: 23727016 PMC3744875

[ref10] Huang Y. Higginson D. S. Hester L. Park M. H. Snyder S. H. (2007). Neuronal growth and survival mediated by eIF5A, a polyamine-modified translation initiation factor. Proc. Natl. Acad. Sci. 104, 4194–4199. doi: 10.1073/pnas.0611609104, PMID: 17360499 PMC1820731

[ref11] Kaech S. Parmar H. Roelandse M. Bornmann C. Matus A. (2001). Cytoskeletal microdifferentiation: a mechanism for organizing morphological plasticity in dendrites. Proc. Natl. Acad. Sci. USA 98, 7086–7092. doi: 10.1073/pnas.111146798, PMID: 11416192 PMC34627

[ref12] Kar R. K. Hanner A. S. Starost M. F. Springer D. Mastracci T. L. Mirmira R. G.. (2021). Neuron-specific ablation of eIF5A or deoxyhypusine synthase leads to impairments in growth, viability, neurodevelopment, and cognitive functions in mice. J. Biol. Chem. 297:101333. doi: 10.1016/j.jbc.2021.101333, PMID: 34688659 PMC8605248

[ref14] Laemmli U. K. (1970). Cleavage of structural proteins during the assembly of the head of bacteriophage T4. Nature 227, 680–685. doi: 10.1038/227680a0, PMID: 5432063

[ref15] Leuner B. Gould E. (2010). Structural plasticity and hippocampal function. Annu. Rev. Psychol. 61, 111–140. doi: 10.1146/annurev.psych.093008.100359, PMID: 19575621 PMC3012424

[ref16] Mandal A. Mandal S. Park M. H. (2014). Genome-wide analyses and functional classification of proline repeat-rich proteins: potential role of eIF5A in eukaryotic evolution. PLoS One 9:e111800. doi: 10.1371/journal.pone.0111800, PMID: 25364902 PMC4218817

[ref17] McLean I. W. Nakane P. K. (1974). Periodate-lysine-paraformaldehyde fixative. A new fixation for immunoelectron microscopy. J. Histochem. Cytochem. 22, 1077–1083. doi: 10.1177/22.12.1077, PMID: 4374474

[ref18] Müller A. Stellmacher A. Freitag C. E. Landgraf P. Dieterich D. C. (2015). Monitoring astrocytic proteome dynamics by cell type-specific protein labeling. PLoS One 10:e0145451. doi: 10.1371/journal.pone.0145451, PMID: 26690742 PMC4686566

[ref19] Nishimura K. Lee S. B. Park J. H. Park M. H. (2012). Essential role of eIF5A-1 and deoxyhypusine synthase in mouse embryonic development. Amino Acids 42, 703–710. doi: 10.1007/s00726-011-0986-z, PMID: 21850436 PMC3220921

[ref20] Padgett L. R. Shinkle M. R. Rosario S. Stewart T. M. Foley J. R. Casero R. A. Jr.. (2023). Deoxyhypusine synthase mutations alter the post-translational modification of eukaryotic initiation factor 5A resulting in impaired human and mouse neural homeostasis. HGG Adv. 4:100206. doi: 10.1016/j.xhgg.2023.100206, PMID: 37333770 PMC10275725

[ref21] Park M. H. (2006). The post-translational synthesis of a polyamine-derived amino acid, Hypusine, in the eukaryotic translation initiation factor 5A (eIF5A). J. Biochem. 139, 161–169. doi: 10.1093/jb/mvj034, PMID: 16452303 PMC2494880

[ref22] Park M. H. Joe Y. A. Kang K. R. (1998). Deoxyhypusine synthase activity is essential for cell viability in the yeast *Saccharomyces cerevisiae**. J. Biol. Chem. 273, 1677–1683. doi: 10.1074/jbc.273.3.1677, PMID: 9430712

[ref23] Park M. H. Nishimura K. Zanelli C. F. Valentini S. R. (2010). Functional significance of eIF5A and its hypusine modification in eukaryotes. Amino Acids 38, 491–500. doi: 10.1007/s00726-009-0408-7, PMID: 19997760 PMC2829442

[ref24] Park M. H. Wolff E. C. Folk J. E. (1993). Is hypusine essential for eukaryotic cell proliferation? Trends Biochem. Sci. 18, 475–479. doi: 10.1016/0968-0004(93)90010-K 8108861

[ref25] Pielot R. Smalla K. H. Müller A. Landgraf P. Lehmann A. C. Eisenschmidt E.. (2012). SynProt: a database for proteins of detergent-resistant synaptic protein preparations. Front Synaptic Neurosci. 4:1. doi: 10.3389/fnsyn.2012.00001, PMID: 22737123 PMC3382120

[ref26] Runge K. Cardoso C. de Chevigny A. (2020). Dendritic spine plasticity: function and mechanisms. Front. Synapt. Neurosci. 12:36. doi: 10.3389/fnsyn.2020.00036, PMID: 32982715 PMC7484486

[ref27] Saini P. Eyler D. E. Green R. Dever T. E. (2009). Hypusine-containing protein eIF5A promotes translation elongation. Nature 459, 118–121. doi: 10.1038/nature08034, PMID: 19424157 PMC3140696

[ref28] Schaffner W. Weissmann C. (1973). A rapid, sensitive, and specific method for the determination of protein in dilute solution. Anal. Biochem. 56, 502–514. doi: 10.1016/0003-2697(73)90217-0 4128882

[ref29] Shin B.-S. Katoh T. Gutierrez E. Kim J. R. Suga H. Dever T. E. (2017). Amino acid substrates impose polyamine, eIF5A, or hypusine requirement for peptide synthesis. Nucleic Acids Res. 45, 8392–8402. doi: 10.1093/nar/gkx532, PMID: 28637321 PMC5737446

[ref30] Suzuki T. Tian Q. B. Kuromitsu J. Kawai T. Endo S. (2007). Characterization of mRNA species that are associated with postsynaptic density fraction by gene chip microarray analysis. Neurosci. Res. 57, 61–85. doi: 10.1016/j.neures.2006.09.009, PMID: 17049655

[ref31] Wang Y. Wang C. Ranefall P. Broussard G. J. Wang Y. Shi G.. (2020). SynQuant: an automatic tool to quantify synapses from microscopy images. Bioinformatics 36, 1599–1606. doi: 10.1093/bioinformatics/btz760, PMID: 31596456 PMC8215930

